# Study of Influencing Factors and the Mechanism of Preparing Triazinedithiol Polymeric Nanofilms on Aluminum Surfaces

**DOI:** 10.3390/ijms11114715

**Published:** 2010-11-18

**Authors:** Fang Wang, Yabin Wang, Yanni Li

**Affiliations:** Northwest Agriculture & Forest University, College of Science, Shaanxi, Yangling 712100, China; E-Mails: 12305031@nwsuaf.edu.cn (Y.W.); lyn0408@nwsuaf.edu.cn (Y.L.)

**Keywords:** triazinedithiol, aluminum surface, functional polymeric nanofilm, electropolymerization mechanism

## Abstract

Triazinedithiol polymeric nanofilm was prepared on a pure aluminum surface by electrochemical polymerization of AF17N. The mechanism of the process was proposed and electrochemical polymerization parameters were investigated. The triazinedithiol polymeric nanofilm had notable lubricity, high dielectric property and superhydrophobic property due to the allyl and fluoro alkyl groups in the AF17N monomer. The chemical structure of poly (6-(*N*-allyl-1,1,2,2-tetrahydroperfluorodecyl)amino-1,3,5-triazine-2,4-dithiol monosodium) nanofilm (PAF17) was investigated by analysis of FT-IR spectra and X-ray photoelectron spectroscopy (XPS). The optimal conditions for the preparation process were based on the data of film weight and thickness. The optimal parameters of monomer concentration, electropolymerization time and temperature were 5 mM, 6 min and 15 °C, respectively. The electropolymerization mechanism was a radical polymerization reaction. It is expected that this technique will be applied in industrial fields for aluminum and aluminum alloy to achieve functional surfaces.

## 1. Introduction

Since the publication of Mori K’s results on the corrosion protection property of triazinedithiols (TDTs) on copper surfaces [1], adsorption and polymerization of TDTs on metal surfaces have received increasing attention in the past few years. The research showed that polymeric nanofilms prepared by different TDTs have a basic anti-corrosion property [2]. Thus, during the last decade, there were many studies concerning other applications of TDTs polymeric film on various metal substrates [3–5]. Monomers of triazinedithiols can be polymerized on metal surfaces by mechanochemical [6], photochemical [7], thermochemical [8], electrochemical [9] or evaporating [10] methods. The electropolymerization process offers the advantages of simultaneous adsorption and polymerization on a metal surface in monomer-electrolyte solution, and the possibility of controlling their chemical and physical properties by changing electrochemical parameters (e.g., current density, potential, monomer concentration, supporting electrolyte, *etc.*). Therefore, electropolymerization has become the mainstream technology for fabricating nanofilms of triazinedithiols. Triazinedithiol molecules with allyl and fluoro alkyl groups in their structure have been studied for many years, because these polymeric nanofilms have notable lubricity [6], high dielectric [11,12] and hydrophobic properties [13,14].

However, the mechanism of the electropolymerization process and the parameters has not been investigated so far. In this paper, we concentrate on the research of the electrochemical mechanism and influencing factors for preparing PAF17 nanofilm, which is obtained from triazinedithiol monomer with allyl and fluoro alkyl groups.

## 2. Results and Discussion

In order to find the optimal concentration of AF17N, FT-IR spectra and X-ray photoelectron spectroscopy (XPS) spectra of PAF17 nanofilms obtained by electropolymerization for five minutes at 25 °C were studied. Then, the electropolymerization time under the optimal concentration was discussed by FT-IR spectra and atomic force microscopy (AFM). Finally, the effect of temperature on the weight and thickness of PAF17 nanofilm was discussed. The current density during the process was kept at 0.3 mA/cm^2^ [13].

### 2.1. Effect of Monomer Concentration on PAF17 Nanofilm

**Figure 1.**
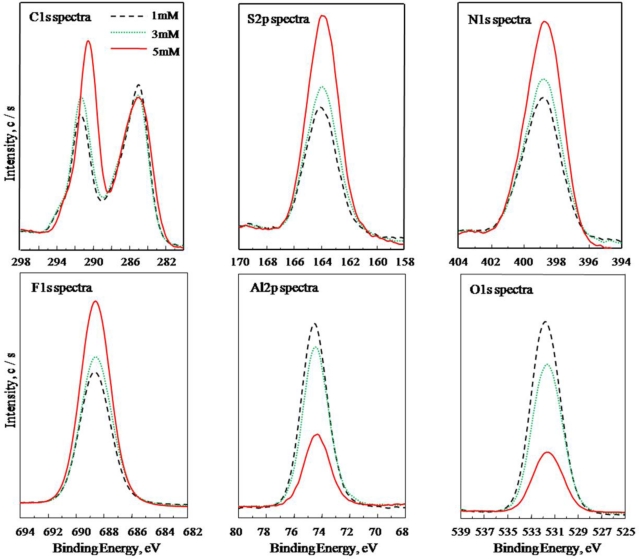
Effect of AF17N concentration on element intensity in the PAF17 nanofilm by XPS analysis. Electropolymerization conditions (Current density: 0.3 mA/cm^2^; Concentration of AF17N: 1, 3, 5 mM; Electropolymerization temperature: 25 °C; Electropolymerization time: 5 min).

In order to study the optimal electropolymerization concentration of AF17N, the atomic concentrations of PAF17 nanofilm were investigated by XPS. Figure 1 shows the XPS data of PAF17 nanofilm polymerized under different AF17N concentrations. It can be seen that the atomic concentrations of C1s, S2p, N1s and F1s on aluminum surface increase with increasing monomer concentration, while the atomic concentrations of Al2p and O1s decrease. The migration rate of ions to the electrode will slow down when the monomer concentration is low. With the monomer concentration increasing, the anions near the aluminum electrode become sufficient for electropolymerization to form the thicker PAF17 nanofilm. However, the micelles will form as the monomer concentration is over 5 mM due to higher molecular weight of the monomer, leading to a decrease of the reactional anions. The result preliminarily suggests that the optimal concentration of AF17N is 5 mM.

**Figure 2.**
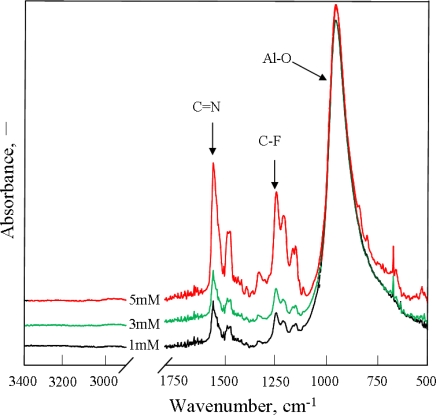
FT-IR spectra of PAF17 nanofilm obtained under different AF17N concentrations.

FT-IR spectra of PAF17 nanofilm obtained using different concentrations are shown in Figure 2. It is noted that when the AF17N concentration is up to 5 mM, the >C=N- and C-F absorption peaks reach the maximum intensities, which also indicates that the optimal concentration of AF17N is 5 mM. Based on the above XPS analysis, the electropolymerization rate of AF17N was slower while the oxidation reaction of aluminum was faster due to the lower monomer concentration. Therefore, the PAF17 nanofilm on an aluminum surface was so thin that the detected >C=N- and C-F absorption in the FT-IR spectra was very weak.

### 2.2. Effect of Electropolymerization Time on PAF17 Nanofilm

**Figure 3.**
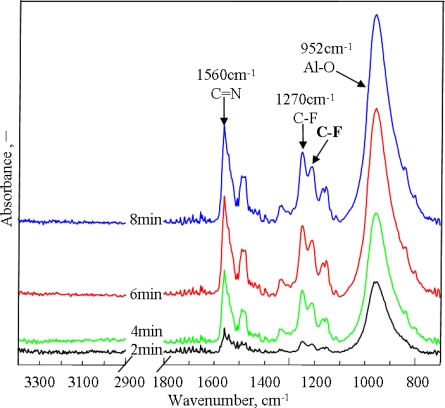
FT-IR spectra of PAF17 nanofilm obtained using different electropolymerization times. Electropolymerization conditions (Current density: 0.3 mA/cm^2^; Concentration of AF17N: 5 mM; Electropolymerization temperature: 25 °C; Electropolymerization time: 2, 4, 6, 8 min).

Figure 3 shows FT-IR spectra of PAF17 nanofilm obtained by using different electropolymerization times. With electropolymerization times ranging from 2 to 6 min, the intensities of >C=N- and C-F absorption peaks become stronger. When the electropolymerization time is more than 6 min, the >C=N- and C-F absorption peak intensities vary slightly. It can be speculated that the PAF17 nanofilm obtained by electropolymerization for 6 min is more compact and insulated, which could depress the further formation of PAF17 nanofilm and the depolymerization reaction could happen.

**Figure 4.**
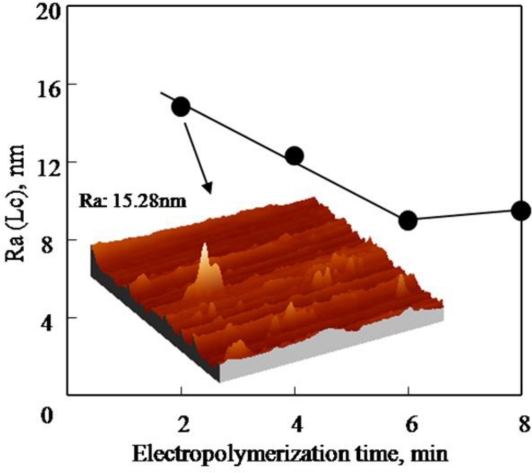
Effect of electropolymerization time on the three-dimensional morphology of PAF17 nanofilm.

The effect of electropolymerization time on the three-dimensional morphology of PAF17 nanofilm is shown in Figure 4. The roughness (Ra) of PAF17 nanofilm decreases when the electropolymerization time is prolonged. However, when the electropolymerization time is over 6 min, the value of Ra shows little increase, which indicates that the PAF17 nanofilm obtained at 6 min is the most uniform. With increasing electropolymerization time, the peeling off of PAF17 nanofilm happens and the nanofilm becomes loose due to the depolymerization reaction. Based on the above analysis, we can conclude that the optimal electropolymerization time is 6 min.

### 2.3. Effect of Electropolymerization Temperature on PAF17 Nanofilm

**Figure 5.**
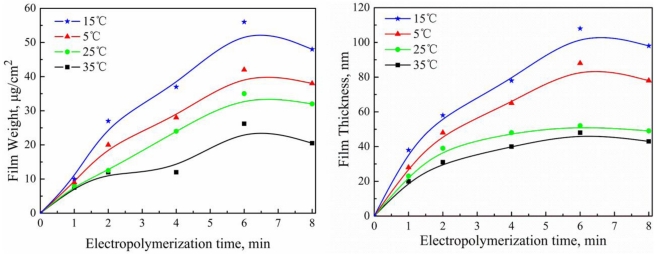
Effect of electropolymerization temperature on the film weight and thickness of PAF17 nanofilms. Electropolymerization conditions (Current density: 0.3 mA/cm^2^; Concentration of AF17N: 5 mM; Electropolymerization temperature: 5, 15, 25 35 °C; Electropolymerization time: 1, 2, 4, 6, 8 min).

The effect of electropolymerization temperature on the thickness and weight of PAF17 nanofilm is shown in Figure 5. When the electropolymerization temperature is 5 °C, the value of thickness and weight is low. It was presumed that the anion of triazinedithiol monosodium in the solution could diffuse to the aluminum surface very slowly, which influenced the rate of the electropolymerization reaction. According to law of thermodynamics, we know that the diffusion rate of a monomer anion depends on temperature. Therefore, the thickness and weight of PAF17 film becomes larger when the electropolymerization temperature is increased from 5 to 15 °C. However, an increase in temperature from 15 to 35 °C, the thickness and weight of PAF17 film appears to decrease. The oxidative reaction and stripping of aluminum electrode could be accelerated, which led to the formation of an alumina layer and pinhole. It was assumed that the alumina layer was insulated, hindering the polymerization of triazinedithiol monosodium.

### 2.4. Electropolymerization Mechanism

FT-IR spectra measurements were performed by reflection absorption to study the chemical structure of PAF17 nanofilm. Figure 2 and Figure 3 show FT-IR spectra of polymeric film obtained by electropolymerization of AF17N under different conditions. The presence of the triazine ring is confirmed by absorption peaks at 1481 cm^−1^, 1536 cm^−1^ and 1560 cm^−1^ due to >C=N- bonds [9,15,16]. Allyl perfluorodecyl amino groups are confirmed by absorption peaks at 1226 cm^−1^, 1250 cm^−1^, 1331 cm^−1^, 1155 cm^−1^ and 1143 cm^−1^ due to C-F stretching vibrations of CF_3_- groups and >CF_2_- groups [12]. However, absorption bands centered at 1645 cm^−1^ and 3090 cm^−1^, 2975 cm^−1^ which are assigned to C=C and =CH_2_ of AF17N monomer, does not appear. It was supposed that allyl groups participated in the electropolymerization reaction [17]. At the same time, the peak of alumina at 956 cm^−1^ was also observed. It was suggested that the electropolymerization of AF17N and oxidation of aluminum occurred simultaneously.

**Figure 6.**
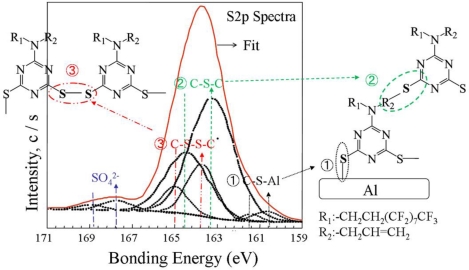
S2p fitted curve of high resolution XPS spectra from PAF17 nanofilm on aluminum plate at 45° tilt degree. Electropolymerization conditions (Current density: 0.3 mA/cm^2^; Concentration of monomer AF17N: 5 mM; Electropolymerization temperature: 15 °C; Electropolymerization time: 6 min).

To confirm the chemical structure of PAF17 nanofilm prepared on an aluminum plate, the S2p fitted curve of XPS spectra was investigated (Figure 6). The S2p spectra consists of peaks assigned to S-Al groups at 160.3 eV, C-S-C groups at 163.5 eV, C-SS-C groups at 163.6 eV and SO_4_^2−^ groups at 167.7 eV. Peak based on S-Al groups indicates the reaction between SH groups and the aluminum substrate during electropolymerization. Peaks based on C-S-C groups suggest the reaction between SH and allylic groups in AF17N during electropolymerization. Peak for the C-SS-C group reveals the electrochemical reaction of thiols. From the S2p XPS data, the PAF17 nanofilm prepared on aluminum plate are confirmed to consist of poly(6-(Nallyl-1,1,2,2-tetrahydroperfluorodecyl)amino-1,3,5-triazine-2,4-disufide). It is assumed that AF17N monomer is dissolved as dithiolate anions in the electrolyte aqueous solution and the dithiolate anions transfer two electrons to the anode (aluminum plate) to change to bisthiyl radicals .Then the bisthiyl radicals cause coupling with each other to yield PAF17 film on the aluminum surface. Besides, lots of bubbles could be observed on the aluminum surface and irritant gas could be smelt simultaneously, which suggested that NaNO_2_ also participated in the polymerization reaction. Based on the above experimental facts, the reaction mechanism is proposed as follows (Equations 1–6):

## 3. Experimental Section

### 3.1. Experimental Materials

**Figure 7.**
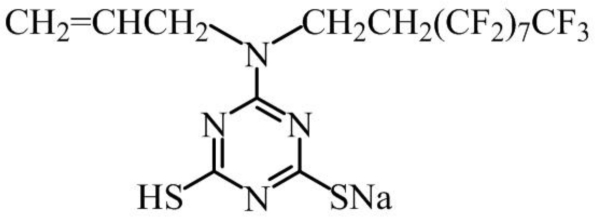
Structure of AF17N monomer.

Test specimens (50 × 30 × 0.1 mm) of pure aluminum (purity no less than 99.9995%) were prepared by cutting a large plate into pieces. All test plates were degreased by ultrasonic washing in acetone for 15 min, then were dried in nitrogen air. 6-(*N*-allyl-1,1,2,2-tetrahydroperfluorodecyl) amino-1,3,5-triazine-2,4-dithiol monosodium (AF17N) was prepared by reaction between 6-(*N*-allyl-1,1,2,2-tetrahydroperfluorodecyl)-amine-1,3,5-triazine-2,4-dichloride and NaSH, according to the method described previously [18]. The structure of AF17N is shown in Figure 7. All of the chemicals were employed as analytical reagents (AR) without further purification. Distilled water was used as solvent, and sodium nitrite (NaNO_2_) was applied as supporting electrolyte. The concentration of supporting electrolyte was kept at 0.15 M.

### 3.2. Preparation of PAF17 Nanofilm under Different Electropolymerization Conditions

The electropolymerization of AF17N was performed by using an electrochemical measurement apparatus (Hokuto Denkou Co. Ltd., HD-3000). The electrolytic cell was equipped with working electrode (aluminum surfaces), Pt counter electrode and reference electrode (saturated calomel electrode, SCE), then was filled with electrolytic solution containing AF17N. Electropolymerization of the AF17N monomer was performed galvanostatically with a current density of 0.30 mA/cm^2^ [13]. Concentrations of AF17N monomer at 1, 3, 5 mM were investigated. Electropolymerization time was 2, 4, 6, 8 min according to our previous study [12].

### 3.3. Characterization

An electronic balance with measurement accuracy of 0.01 mg (CP225D, Sartorius) was used to investigate the polymeric film weight. Film thickness was measured by using a JASCO M-150i ellipsometer (Jasco Tokyo Japan). FT-IR spectra were carried out using JASCO IR-5500 (Jasco Tokyo Japan) by high-performance reflection absorption spectroscopy (RAS). A reflection attachment was used at an incident angle of 80° together with a wire grid polarizer. X-ray photoelectron spectroscopy (XPS) was performed to investigate the elemental composition of aluminum surface. Spectra were obtained by using a ULVAC PHI-5600 spectrometer equipped with monochrome Al Kα radiation (1,486.6 eV). The pressure in the preparation chamber was less than 10^−7^ Torr and less than 4 × 10^−10^ Torr in the analysis chamber. Samples were examined over an area of 800 × 2000 μm, and photoelectron spectra were recorded with a take-off angle of 45°. The treated substrate was observed by atomic force microscopy (AFM) (Nanoscope III Scanning Probe Microscope, Digital Instruments, Veeco Metrology Group) with contact mode to characterize the three-dimensional morphology.

## 4. Conclusions

PAF17 nanofilm was successfully prepared on an aluminum surface by galvanostatical electropolymerization of AF17N with confirmation of FT-IR and XPS spectra. The film thickness and weight reached a maximum at 15 °C. The optimal parameters of monomer concentration and electropolymerization time were 5 mM and 6 min. The electropolymerization mechanism was a radical polymerization reaction, and the allyl and thiol groups in AF17N monomer were involved in the electropolymerization forming functional polymeric nanofilm. It is expected that this technique will be applied in preparation of lubricating, dielectric and hydrophobic surfaces on aluminum substrates.

## Figures and Tables

**Scheme 1.**
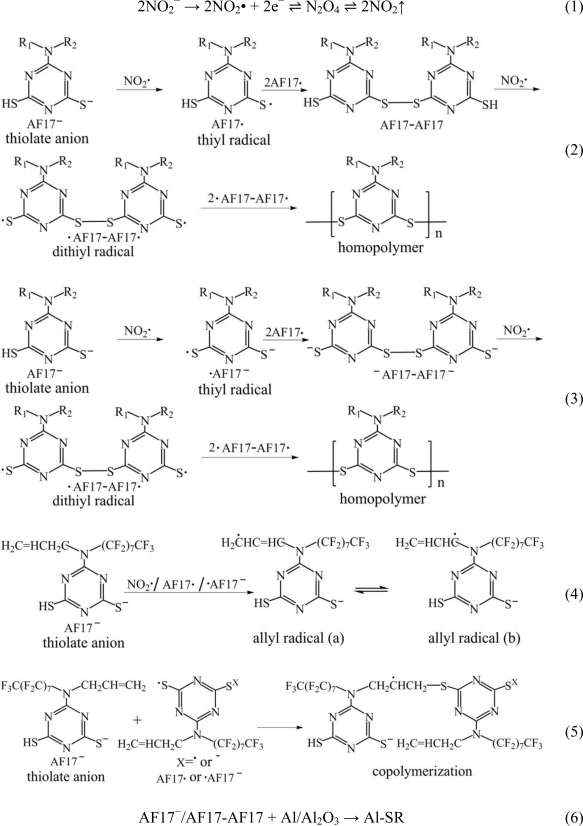
Radical mechanism for the formation of PAF17 nanofilms on aluminum surface in the aqueous solution of AF17N and NaNO_2_ electrolyte.
